# Environmental Lighting Conditions, Phenomenal Contrast, and the Conscious Perception of Near and Far

**DOI:** 10.3390/brainsci14100966

**Published:** 2024-09-26

**Authors:** Birgitta Dresp-Langley, Adam J. Reeves

**Affiliations:** 1 Centre National de la Recherche Scientifique, UMR 7357, Strasbourg University, 67000 Strasbourg, France; 2 Psychology Department, Northeastern University, Boston, MA 02115, USA; a.reeves@northeastern.edu

**Keywords:** achromatic vision, ON–OFF visual pathways, spatial contrast, non-linear integration, visual adaptation, subjective contrast, assimilation, perceptual ambiguity

## Abstract

Background: Recent evidence in systems neuroscience suggests that lighting conditions affect the whole chain of brain processing, from retina to high-level cortical networks, for perceptual and cognitive function. Here, visual adaptation levels to three different environmental lighting conditions, (1) darkness, (2) daylight, and (3) prolonged exposure to very bright light akin to sunlight, were simulated in lab to investigate the effects of light adaptation levels on classic cases of subjective contrast, assimilation, and contrast-induced relative depth in achromatic, i.e., ON–OFF pathway mediated visual configurations. Methods: After adaptation/exposure to a given lighting condition, configurations were shown in grouped and ungrouped conditions in random order to healthy young humans in computer-controlled two-alternative forced-choice procedures that consisted of deciding, as quickly as possible, which of two background patterns in a given configuration of achromatic contrast appeared lighter, or which of two foreground patterns appeared to stand out in front, as if it were nearer to the observer. Results: We found a statistically significant effect of the adaptation levels on the consciously perceived subjective contrast (F(2,23) = 20.73; *p* < 0.001) and the relative depth (F(2,23) = 12.67; *p* < 0.001), a statistically significant interaction between the adaptation levels and the grouping factor (F(2,23) = 4.73; *p* < 0.05) on subjective contrast, and a statistically significant effect of the grouping factor on the relative depth (F(2,23) = 13.71; *p* < 0.01). Conclusions: Visual adaption to different lighting conditions significantly alters the conscious perception of contrast and assimilation, classically linked to non-linear functional synergies between ON and OFF processing channels in the visual brain, and modulates the repeatedly demonstrated effectiveness of luminance contrast as a depth cue; the physically brighter pattern regions in the configurations are no longer consistently perceived as nearer to a conscious observer under daylight and extreme bright light adapted (rod-saturated) conditions.

## 1. Introduction

For consciously perceiving meaningful global structure and form in a world composed of complex visual scenes, the human brain relies on multiple stages of visual processing. At the post-retinal level, incoming signals are fed into cells with antagonistic ON–OFF center-surround function. These cells selectively process luminance increments and decrements and provide input to two parallel channels, the so-called ON and OFF pathways [1]. These pathways receive, transform, and transmit signals of light and contrast in the first stage of visual integration that is critical to our conscious perception of complex objects and their phenomenal contrast properties, because it sets the stage for all further coding of spatial configuration, including the conscious perception of surfaces in the plane appearing nearer or further away from the observer [2,3], enabling a meaningful scenic interpretation. Seminal psychophysics from about 40 years ago [4] have led us to understand that the visual brain transforms contrast signals in a profoundly nonlinear manner [4], fulfilling a complex function that pools differences in contrast across multiple surfaces in a scene rather than responding to local contrast as such [4,5,6]. In neurophysiology, such non-linearity is reflected by complex functional synergies between the ON and OFF pathways [1,5,6,7]. The two pathways have been classically thought to signal independently in a mirror-symmetric fashion; however, recent neuroscience has clarified that this view is not sustainable [6,7]. Both pathways interact and cooperate when needed, contributing synergistically to conscious visual perception [6]. These functional synergies are profoundly non-linear, as earlier psychophysics have concluded [4,5,6]. They show pronounced light–dark asymmetries that cannot be linked to any simple (i.e., directly mirror-symmetric, summative, multiplicative, etc.) function of local contrast [4,7]. Some of this non-linearity originates at the cellular level and is then preserved at all further stages of processing [7]. This is believed to enable the visual brain to function consistently by adaptation to a wide variety of luminance conditions, from photopic to low mesopic light, for the transformation of complex spatiotemporal contrast into a meaningful conscious percept [4,7].

**Figure 1:**
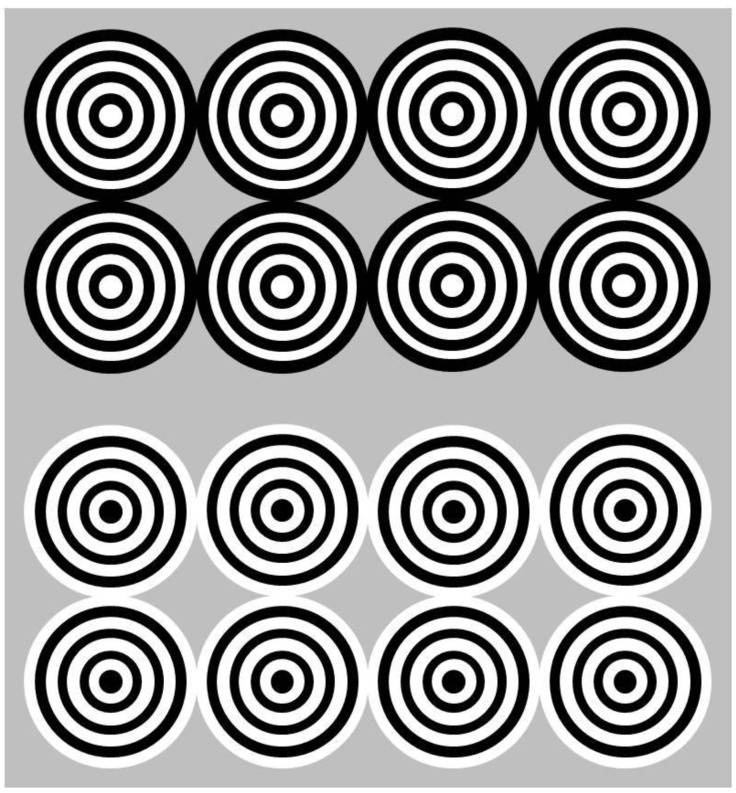
In conscious perception, the “pincushion”-shaped surfaces surrounded by black and white circular surfaces forming the eight disks in the top row configuration look phenomenally darker than the grey background; those in the bottom row are perceived as lighter. Paradoxically, when asked to adjust the physical luminance of the central surfaces to that of the background, observers systematically adjust both test surfaces (the “pincushions”) to a darker luminance. This inconsistency between physical luminance matching and the conscious perception of phenomenal contrast and assimilation points towards complex functional pathway interactions in the visual brain.

In psychophysics, ON and OFF pathway synergies have classically been linked to effects of phenomenal contrast and assimilation within a from-low-level-to-conscious processing chain of achromatic spatial contrast [1,2,3]. In such phenomena, the perceived intensity of a test field increases by comparison with the intensity of a reference field or background (phenomenal contrast), or where the perceived intensity of the test approaches that of the reference (phenomenal assimilation) or background [8,9,10,11,12,13,14]. The effects are highly ambiguous, and sometimes the psychophysically measured data are inconsistent with the conscious percept. For example, in Figure 1, the small pincushion-shaped surface surrounded by four disks made of black and white rings on a uniform grey background looks phenomenally darker (Figure 1, upper row) when the nearest surrounding arcs are black and lighter (Figure 1, bottom row) when the nearest surrounding arcs are white. The phenomenally darker “pincushions” in the top row are seen further away in depth compared with the phenomenally lighter ones in the bottom row. While the phenomenally darker “pincushion” surface consistently requires a psychophysically matched luminance that is lower than that of the reference background (assimilation), the phenomenally lighter one, paradoxically, is also matched to a lower physical luminance [14]. Similar observations in other configurations, reported earlier in terms of Hamada’s brightness paradox [9,10,15] point towards functional antagonism between early and higher-level visual mechanisms in the conscious perception of complex achromatic patterns. In addition, phenomenal contrast is often accompanied by other conscious sensations: the “pincushion” here (Figure 1) may subjectively appear nearer or further away from the conscious observer.

Local characteristics of multiple surface displays have a limited impact on the mechanisms that adaptively transform complex spatiotemporal contrasts in visual scenes for conscious perception. Local contrast polarity, for example, may have an effect or not depending on the type of task [4,16,17,18,19] and on whether a given conscious percept of phenomenal depth or contrast is induced inwardly by a surrounding surface or outwardly by an inner surface towards the surrounding field [11,12,16,17,18,19,20,21]. Some studies [16,17] demonstrate effects of local contrast polarity on outwardly induced phenomenal contrast and “nearer” versus “further away” percepts in colored and achromatic surfaces on gray backgrounds in a mixed-block-three-alternative forced-choice procedure. No effects of contrast polarity were found in configurations with inwardly induced phenomenal contrast or assimilation [18,20], or inwardly induced phenomenal depth in terms of conscious percepts of “nearer” versus “further away” [19,21]; these latter are generally strongest for the highest positive or negative contrast in planar multi-surface configurations [22]. Multisensory testing had shown that the highest positive or negative contrasts produce the fastest response times for “nearer”, especially when immediately preceded by a brief 200–100 Hz pure tone signal [23]. There is no simple linear relationship between luminance contrast intensity and phenomenal contrast or assimilation, which are strongest in configurations with lower contrast intensities of positive or negative polarity [4,11,12] and with brief display exposure durations of only 10 milliseconds [24]. This crucial finding highlights their early genesis in the visual for deterministic conscious perception, where black and white are highly dynamic antagonistic forces rather than the end points of any single stimulus continuum. This view of light–dark antagonism, as pointed out by Gerald Westheimer in his review paper [1], preceded visual science by decades of physical investigation into energy absorption and photochemical activity changes with varying incident light levels, which finally led to the discovery of the ON and OFF channels in the visual brain.

Changes in visual adaption as a result of changes in environmental lighting levels matter to conscious visual perception [25,26,27]. The hypothesis that lighting conditions and the resulting visual adaptation levels modulate higher order brain activity and influence interactions between pathways from retina to cortical processing harks back to ideas already formulated in Helson’s seminal book published in 1964 [25]. Recently, novel lines of investigation in systems neuroscience have shown that lighting conditions could alter a wide range of higher-order brain activities [26,27], with functional interactions originating at the receptor level, some of them preserved at all further stages of processing including those underlying conscious perception [26,27]. Changes in environmental lighting conditions, to which the human visual system functionally has to adapt, include transitions from darkness to daylight and vice versa or transient changes in visual adaptation following exposure to very bright light, which produces the temporary selective saturation of visual receptors filtering contrast input from the environment. The goal of the present study was to investigate the extent to which three different conditions of visual adaptation, (1) dark-adapted vision, (2) normal daylight vision, and (3) sustained bright light, inducing rod saturation, affect outwardly induced phenomenal contrast and assimilation, and the related conscious perception of “nearer” in planar achromatic multi-surface pattern configurations. Some of these are similar to configurations used in previous studies [17,22]. Here, pure trial blocks with exclusively achromatic signal input and two-alternative forced choice tasks testing for phenomenal contrast/assimilation and for conscious sensations of “nearer” were presented. Whether symmetry [28] or lack thereof affects the conscious perception of either is not known, but it has been stressed that the presence of structural regularities in the visual scene matter [17,22,29]. The multiple-surface configurations, presented to the left and to the right of a central fixation point that helped the observer maintain attention, contain exactly the same structural regularities, without being geometrically mirror-symmetric.

## 2. Materials and Methods

Tests were run on a PC computer equipped with a mouse device and a high-resolution color monitor. Selective combinations of RGB increments generating the colors of the stimuli were calibrated with a spectrophotometer (Cambridge Research Instruments, Hopkinton, MA, USA). Participants were comfortably seated at the console at a distance of 75 cm from the screen, their heads comfortably resting on a head-and-chin support.

### 2.1. Participants

Eight individuals, most of them undergraduate students at Northeastern University, Boston USA (NU), with normal or corrected-to-normal vision participated in the simultaneous contrast task. Eight other observers, most of them also undergraduate students at NU with normal or corrected-to-normal vision were run in the relative distance task. Individuals participated in the context of their course requirements and, although naïve to the goal of the study, they were all psychophysically literate. This entails being aware that their responses are not used to judge performance, that focused attention and swift response without thinking too much is necessary, there are no “right” or “wrong” answers, and that what is tested for is a “perceptual sensation without logical or analytical thinking about what is going on”. All students provided informed consent to participate in compliance with the national and international ethical standards for experimentation on human observers when the study was performed. The experiments were performed in conformity with the ethical rules and recommendations of the Helsinki Declaration for experimentation on humans. Approval of an ethics board was not required. Signing up for the computer testing was part of the NU undergraduate course requirements at the time, and ethics board approval was waived the test protocol being assimilated to a survey procedure.

### 2.2. Pattern Luminance Calibration

**Figure 2:**
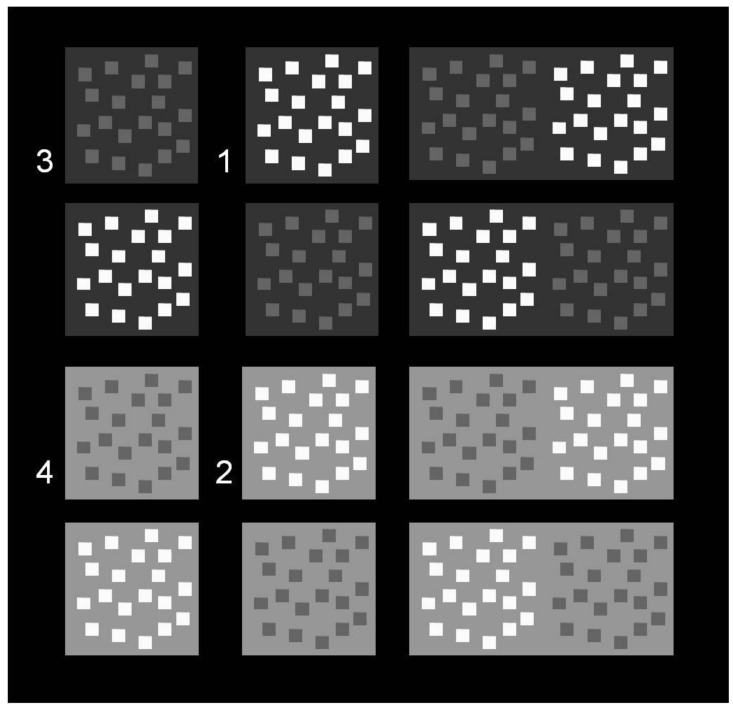
Spatial pattern configurations with brighter and darker pattern elements on light and dark grey backgrounds. The bright and dark pattern elements make their immediate backgrounds appear either phenomenally darker or phenomenally lighter, although the two backgrounds in a given display have exactly the same physical luminance. Configurations were presented ungrouped (left) and grouped (right) on a black general background. The relative position of brighter and darker pattern elements on the left or right in a configuration varied randomly across presentations. Note that the configurations were always paired in a given trial, with one presented to the left of fixation, the other to the right. As we can see in Figure 2, configuration 1 was always paired with configuration 3; configuration 2 was always paired with configuration 4.

The patterns consisted of pairs of spatially separated configurations or single regrouped configurations, with twenty square-shaped achromatic pattern elements placed on light grey and dark grey background fields, displayed on a black (2.45 cd/m^2^) general background (Figure 2). In the ungrouped configurations, the horizontal distance between two grey backgrounds on the black screen was 4 cm. A given configuration on the left or right in the ungrouped configuration trials was 1.5 cm away from the central fixation mark that appeared between trials. The height of each grey background square was 9.7 cm, and the width was 10 cm. The smallest horizontal distance between pattern elements on a grey background was 0.4 cm, and the smallest vertical distance was 0.5 cm. All small pattern elements had identical heights (0.9 cm) and widths (1 cm). The luminance of the pattern elements was 148.6 cd/m^2^ (R = 190, G = 190, B = 190) for the brighter ones and 54.3 cd/m^2^ (R = 100, B = 100, G = 100) for the darker ones. The luminance of the light grey background was 72.7 cd/m^2^ (R = 150, G = 150, B = 150), and the luminance of the dark grey background was 23.2 cd/m^2^ (R = 50, G = 50, B = 50). The configurations on the light and dark grey backgrounds produced four levels of physical luminance contrast C given by

C = (Lpattern − Lbackground)/(Lpattern + Lbackground)(1)

in terms of the Michelson formula. Numerical values are given in Table 1.

### 2.3. Task Instructions

The brighter pattern elements appeared randomly on the left or the right of a given configuration, with the darker elements on the other side. Two response alternatives (“left” or “right”) were given to subjects in each of the two tasks. In the phenomenal contrast task, they were asked to indicate on which side of a given configuration (“left” or “right”) the grey pattern background appeared brighter to them. It was made clear to all of them that they should judge the relative brightness of the background, not that of the pattern elements. In the phenomenal depth task, subjects had to decide on which side of a given configuration (“left” or “right”) the pattern elements appeared nearer to them.

### 2.4. Measurement Procedure

The tests were run in a room with no windows, under three separate conditions of visual adaptation. In the first (daylight condition), subjects were run under conditions similar to daylight, generated by a diffuse soft white light source (General Electric, Boston, MA, USA). The correlated color temperature of the Tungsten light was about 6700 Kelvin, corresponding to daylight conditions at noon on a clear sunny day without clouds. In the second (dark-adapted condition), subjects were fully dark-adapted for 25 min, and the experiments were run with all lights off in the room.

In the third condition (bright-light induced rod saturation), subjects were pre-exposed, following standard procedure, to an intense full-field white adaptation light from a model PS22 Grass Photonic Stimulator, no longer commercially available in this version, run at 45 Hz, which was progressively intensified to 1,500,000 candelas, where it was held before the subject’s open eyes for one minute, achieving at least 94% of rod bleaching. The rod bleaching was sufficient for the recovery of the rods to be delayed at least 15 min. The fraction of rod bleach was *p* = 94% given exposure to the 1.5 million cd/m^2^ Grass lamp, according to Rushton’s experimental data on rhodopsin bleaching in situ [30]. Subjects were then dark-adapted for two minutes to permit the cones to recover, leaving the rods relatively inactive for the next ten to fifteen minutes. The test trials took three to five min and were run immediately after the 2 min cone recovery period. Thus, in the dark-adapted condition, both the rods and cones were maximally sensitive; in the rod-saturated condition, the cones were sensitive, but the rods, being bleached, were insensitive. We controlled the rods in this manner, because after 20 min. of full dark adaptation, the rods are actively responding to a quite high luminance input [31] and also contribute to peripheral color vision [32]. In each of the conditions, grouped and ungrouped configurations with a given background intensity and pattern element brightness were presented in random order for about one second each. Inter-stimulus intervals typically varied from one to three seconds, placed under the control of the participant to allow for the individually experienced after-images to vanish before the next trial was initiated. Individuals were instructed to blink between trials to check for residual after-images, which were especially noticeable in the dark-adapted condition (after-images were rarely experienced in the daylight and rod-saturated conditions). Between trials, a uniformly dark (2.45 cd/m^2^) screen, with a small, slightly brighter, fixation point displayed in the center was presented. The fixation point was to help individuals maintain the direction of the gaze. Each individual test session consisted of 10 trials per factor level for a 3 × 2 × 2 × 2 Cartesian design, with three adaptation levels, two pattern backgrounds (light versus grey), two types of spatial configuration (grouped versus ungrouped), and two pattern positions in random order (left or right), yielding a total of 240 trials per individual.

### 2.5. Response Coding

Individual responses from trials via the computer keyboard (‘1’ for ‘left’, ‘2’ for ‘right’) were coded and then stored in excel files for data analysis. In the case of the phenomenal contrast task, the responses were coded consistently [5], as ‘contrast’ when the darker pattern elements in the two displays shown made a background look brighter and as ‘assimilation’ when the brighter ones made a background look brighter. In the phenomenal depth task, the ‘left’ (‘1’) and ‘right’ (‘2’) responses indicated in which of two displays the brighter patterns elements were seen as ‘nearer’ in consistency with the contrast depth cue hypothesis. The response probabilities for ‘contrast’, ‘assimilation’, and ‘nearer’ are computed by dividing the number of such responses in a block divided by the total number of trials.

## 3. Results

**Figure 3:**
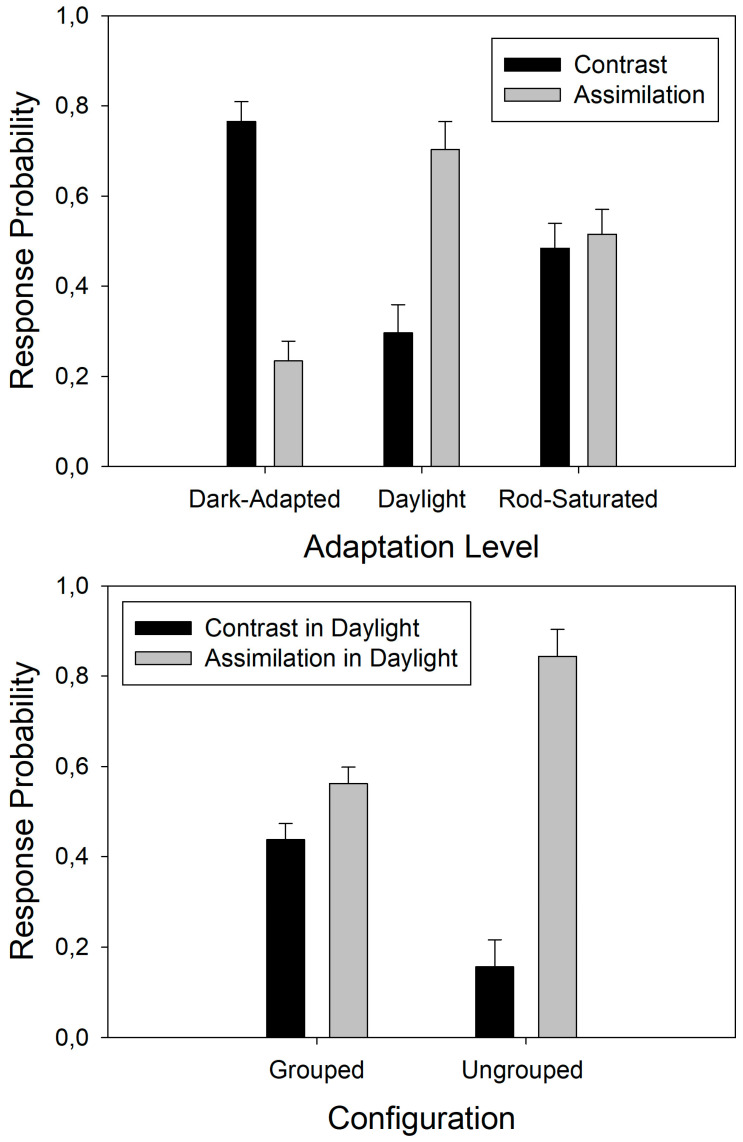
Response probabilities, with error bars, translating the statistically significant effects of the visual adaptation level on the phenomenal contrast and assimilation (**top**). Note: Both are complementary dependent variables in the measurement of this phenomenon; as a consequence, the sum of the two response probabilities (P‘contrast’ + P‘assimilation’) is always 1 for one and the same factor level but not across factor levels. The response probabilities, with error bars, for the statistically significant interaction between subjective contrast and configuration in the daylight condition are shown below (**bottom**).

The original data in terms of the average response probabilities per individual and test condition are available in File S1 of the Supplementary section. The full three-way ANOVA protocols (computer output files) are available in a portable document file under File S2 of the Supplementary section. The response probabilities from the phenomenal contrast task were fed into analysis of variance (three-way ANOVA) for a 3 × 2 × 2 × 2 analysis plan with three adaptation levels (adaptation factor), two pattern background levels (background factor), and two types of spatial configuration (grouping factor). As the two pattern positions (‘left’ and ‘right’) were randomized and counterbalanced, this source of variation was averaged over. The results show a statistically significant effect of the adaptation level (F(2,23) = 20.73; *p* < 0.001). This effect, with standard errors, is plotted here below in Figure 3 (top graph). The statistical analysis further signaled a significant interactive effect of the adaptation level and the grouping factor (F(2,23) = 4.73; *p* < 0.05) on the response probabilities. Neither the grouping (configuration) factor nor the background factor (light backgrounds versus dark backgrounds) produced a statistically significant main effect. Post-hoc testing (Holm–Sidak) permitted determining which comparisons in the interaction between the factors adaptation level and grouping carried significance. The results showed a significant difference between the response probabilities for grouped versus ungrouped configurations within the daylight condition of the adaptation level factor (t(1,1) = 2.71; *p* < 0.05). The effects, with standard errors, for this comparison are also shown here below in Figure 3 (bottom graph). The results clearly showed significantly higher probabilities for ‘contrast’ in the dark-adapted condition and significantly higher probabilities for ‘assimilation’ in daylight (Figure 3, top). The difference between ‘assimilation’ and ‘contrast’ under daylight conditions was significantly stronger in the ungrouped configurations, where three spatially separated background fields were present instead of the two displayed in the grouped condition. Saturating the rods did not alter this grouping effect.

**Figure 4:**
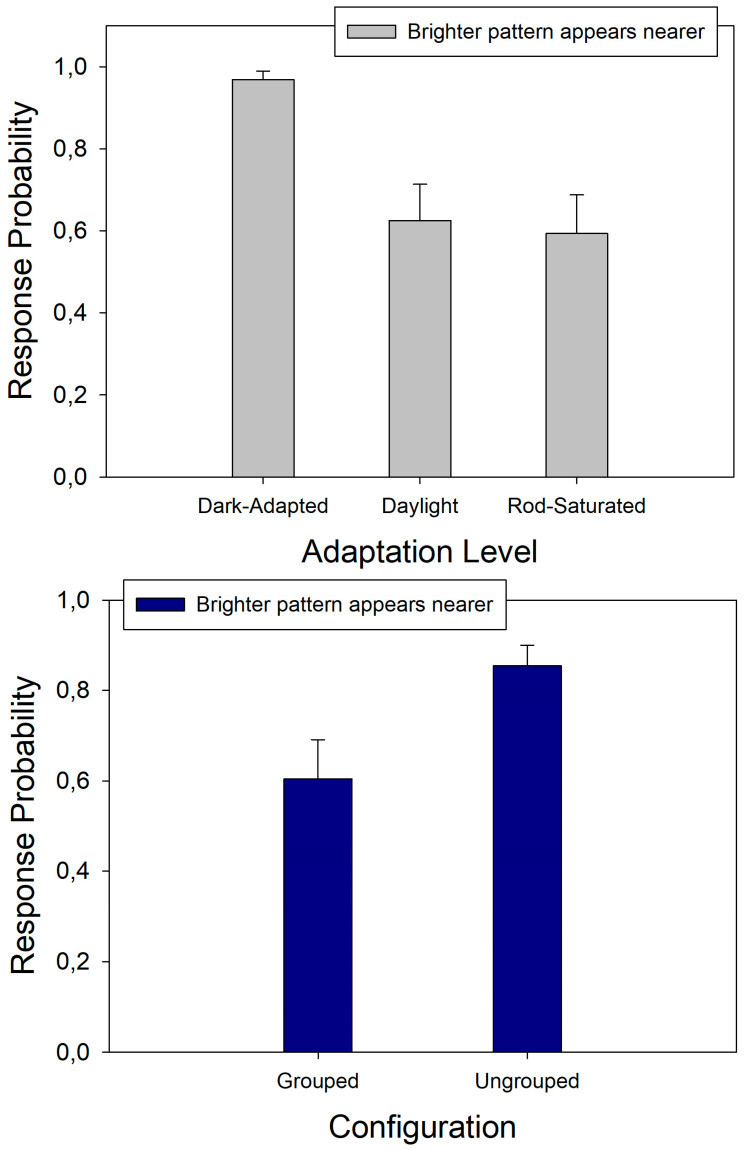
Response probabilities, with error bars, translating the statistically significant effects of the visual adaptation level on the phenomenal depth (**top**) and the significant effects of configuration (**bottom**). There is no significant interaction between the effects of the adaptation levels and the configuration on the subjective depth.

The response probabilities for the brighter patterns’ elements in a given display to be seen as nearer (phenomenal depth task data) were also fed into analysis of variance (three-way ANOVA),with the same 3 × 2 × 2 × 2 analysis plan. This analysis showed a significant effect of the adaptation level (F(2,23) = 12.67; *p* < 0.001) and a significant effect of grouping (F(2,23) = 13.71; *p* < 0.01) but no significant interaction between factors and no significant effect of background luminance. The effects of the adaptation level on the response probabilities for the relative depth are shown here below in Figure 4 (top graph), and the grouping effect or configuration effect is shown below (Figure 4, bottom graph). These results clearly showed that the probability of the brighter pattern elements in a display to be perceived as ‘nearer’ was significantly higher in the dark-adapted condition (Figure 4, top), where the statistically highest probabilities for ‘contrast’ were found (Figure 3, top). Brighter pattern elements were statistically more likely to stand out as ‘nearer’ in the ungrouped configurations, where three spatially separated background fields were present instead of the two displayed in the grouped condition.

**Figure 5:**
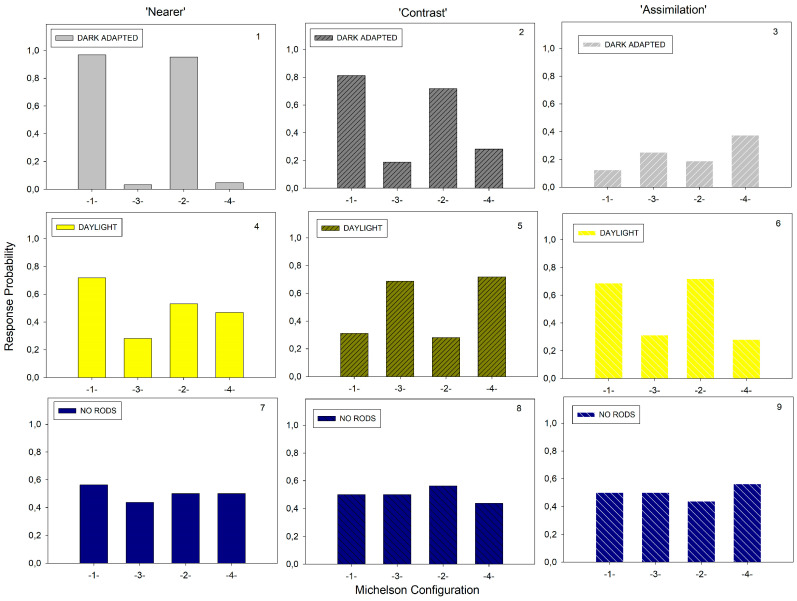
Response probabilities for ‘nearer’, ‘contrast’, and ‘assimilation’, as a function of the adaptation level for each Michelson configuration, labeled ‘1’, ‘3’, ‘2’, and ‘4’, as in Figure 2; the corresponding numerical values (Michelson contrasts) are given in Table 1.

As explained in the Introduction, there is no simple linear relationship between either the polarity/intensity of local luminance contrast and the consciously perceived phenomenal contrast/assimilation. To highlight the influence of the different adaptation levels on these phenomena for each of the physical contrast configurations here, labeled 1, 2, 3, and 4 in Figure 2, with the corresponding Michelson values given in Table 1, we plotted the response probabilities for ‘contrast’, ‘assimilation’, and ‘nearer’ for each Michelson configuration. Note that the configurations were always paired in a given trial, with one presented to the left of fixation and the other to the right. As we can see in Figure 2, configuration 1 was always paired with configuration 3; configuration 2 was always paired with configuration 4 (see also the corresponding columns in the two excel data sheets in File S1 of the ‘Supplementary Materials’ section). The plots are shown in Figure 5 (graphs 1 to 9). The brighter pattern elements were the most likely to be seen as closer to the observer in the dark-adapted condition, with non-ambivalent strong ON–OFF mediated phenomenal contrast effects (Figure 5, comparing graphs 1 and 2). While the configuration producing the strongest phenomenal contrast and “nearer” percepts was, indeed, the one with the strongest (physical) Michelson contrast (Michelson Configuration 1 on the x-axis, referring to Figure 2 and Table 1), the effect of configuration 2 was just as strong; yet, configuration 2 had only half the physical contrast intensity of configuration 1 and less physical intensity than configuration 2. This result is fully consistent with similar observations by Shapley and Gordon [4] of profoundly non-linear achromatic phenomenal contrast in achromatic patterns. In the daylight condition here, the brighter pattern elements were less likely to be seen as closer to the observer compared with the dark-adapted condition. The highest likelihoods for ‘nearer’ in daylight went together with the strongest ON–OFF mediated assimilation (Figure 5, comparing graphs 4 and 6) in daylight; the conscious percepts became altogether more ambiguous. In the rod-saturated condition (Figure 5, graphs 7, 8, and 9), the response probabilities signaled complete ambivalence in the conscious perception of the phenomenal contrast and depth. One might have expected roughly similar results in the daylight and rod-saturated conditions; yet, the effects on phenomenal contrast/assimilation and depth were different in the two conditions. Under daylight, the conscious perception of either was less ambivalent. As the spatial extent of the stimulations here were essentially foveal, any contribution of rods in the retinal periphery under daylight would have been minimal. Recent system neuroscience has shown melanopsin-containing retinal projections towards cortical areas in the primate brain involved in image-related visual processing [26,33]. A contribution of photosensitive melanopsin to the effects under daylight here may explain the differences in results between the daylight and rod-saturated conditions. This, however, is speculation, albeit interesting.

## 4. Discussion

It is shown that the phenomenal contrast and assimilation, observed previously in other complex achromatic, significantly depend on the visual adaption level. Both are complementary aspects of conscious perception, expressing complex and seemingly paradoxical antagonistic effects in the processing of spatial contrast by the ON and OFF pathways from retina to cortex [1]. The significantly higher response probabilities for contrast in the dark-adapted condition is correlated with the significantly higher probability of brighter foreground patterns to be perceived as nearer, validating the contrast as a depth cue hypothesis [2], repeatedly demonstrated under conditions of dark adaptation/mesopic vision [2,3,16]. Under daylight, the effectiveness of the contrast depth cue diminishes; it disappears entirely in the rod-saturated visual adaptation.

The observations here are consistent with previous findings of multiple-surface displays linked to functional synergies of the ON–OFF pathways in the mammalian visual system [1,2,5,6,7]. It has been suggested that these processing channels have evolved to yield rapid information transfer for both increments and decrements of light in complex achromatic stimuli [1,6] and to facilitate contrast processing in ambiguous foreground–background situations, which are omnipresent in the natural environment outside artificially controlled laboratory conditions [7,8,9]. The two pathways respond differently to luminance contrast, depending on the environmental context [7]. It has been suggested that strong illumination levels increase the visual size of light surfaces in nature, the average response increments from ON and OFF cortical pathways then become stronger, and patterns are perceived as brighter. In both animal models and humans, low contrasts drive stronger responses from ON pathways, whereas high contrasts drive stronger responses from OFF pathways [7]. The functional advantages of ON–OFF pathway interactions [6,7] for the conscious perceptual integration of contrast surfaces in complex scenes are many, given that contrast is strongly correlated with multiple light/dark ratios of visual performance in high-level perceptual phenomena and conscious vision [1].

**Figure 6:**
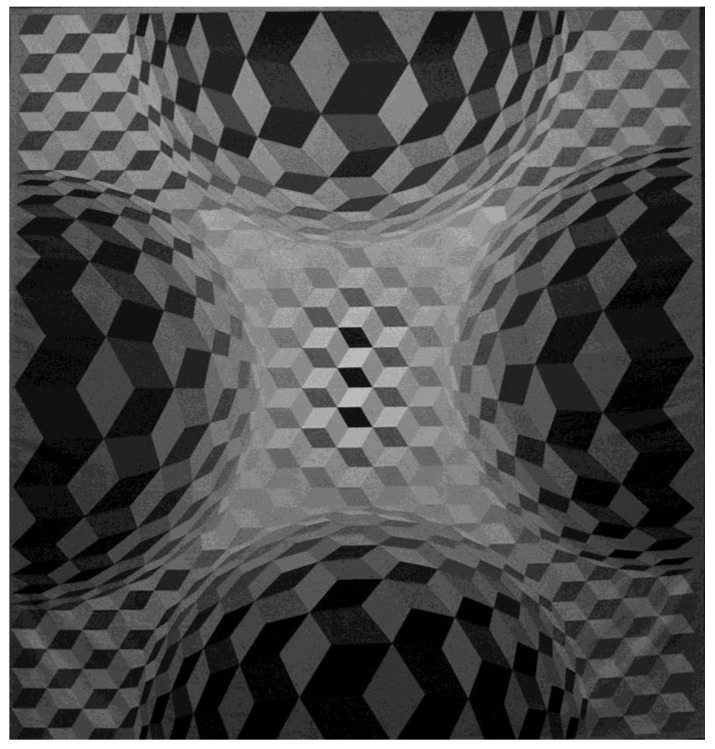
Graphical art reproducing powerful depth effects from 2D simulations of contrast, shape, aerial perspective, relative size, and shading (shadows), as they may occur under natural 3D viewing conditions, under conditions of varying illumination (original photograph taken by the first author, 2013, with permission from The Victor Vasarely Foundation, Aix-en-Provence, France).

The observation that contrast effects are significantly more likely under dark adaptation, while assimilation is more likely in daylight conditions, is explained by the differential pooling of multiple light/dark ratios by the ON–OFF channels [34,35,36,37,38], which affects the relative surface size [35], hence the stronger relative effects of either in the ungrouped conditions, where three spatially separated surfaces are present in a display instead of two in the grouped conditions. Assimilation predominates in the cone-driven light-adapted state, while contrast predominates when the rods are fully active, after complete dark adaptation. Rods feed a scotopic pathway designed to catch quanta, which are rare at night, and so is predominantly or exclusively ‘ON’, whereas cones feed into the photopic pathway which, receiving a surfeit of quanta, can disregard absolute light levels and instead code ratios of light intensities [39]. Possibly the ‘ON’ response to the target patch, being strengthened by dark-adapted rods, increases the probability of contrast and decreases that of assimilation. The effects described here are low-level effects influencing the conscious perception of achromatic displays. Previous research had shown that adaptation levels have no significant effects on similar response probabilities when color information needs to be processed to resolve spatial ambiguities for phenomenal contrast, assimilation, and depth [16,22]. Color involves higher levels of information integration, bearing in mind that the main subcortical pathways serving conscious visual perception are the magnocellular (M) pathways for achromatic vision, to which the ON–OFF system belongs, and the parvocellular (P) channels for integrating color, which require longer visual processing times at higher levels of functional integration [37,38,39]. The relative contribution of P and M pathways to spatial vision is a long-standing and still unresolved issue, given that the P pathways also receive input from ON- and OFF-dominated receptors [37,38,40]. According to the luminance contrast as a depth cue hypothesis [2], the pattern elements with the stronger luminance contrast, i.e., the objectively brighter ones, should always be seen as nearer. The results from the experiments here show that this is confirmed to be the case under dark-adapted conditions but not when the adaptation level changes. In daylight, the conscious depth percepts become more ambiguous, accompanied by a significant decrease in phenomenal contrast. In the rod-saturated condition, all the conscious percepts tested for become entirely ambiguous regardless of the physical contrast of the displays. The luminance contrast as a depth cue hypothesis [2] is based on mesopic-vision laboratory experiments. In natural viewing, all surfaces physically nearer or further away from the observer may be overshadowed, depending on the angle of the sunlight on a very bright and clear day or the lighting conditions on a grey, overcast, and cloudy day. These factors modulate the effectiveness of the luminance contrast as a depth cue in natural viewing. Further research may help better identify the viewing conditions in which brighter surfaces are consistently seen as further away. Phenomenal examples of such cases can be found in landscape paintings by Corot and Turner and even more so in Victor Vasarely’s work [17], as shown here below in one of his compelling pieces (Figure 6). Paintings and visual art are powerful probes to brain processes underlying conscious perception [41,42,43,44]. When artists deploy specific effects to obtain a pictorial or graphic representation of what they consciously perceive (Figure 6), they are performing an experiment [41,45,46,47]. The first stage that underlies such conscious experimentation is the one that enables us to differentiate between light and dark signals in the physical world via complex antagonistic functions from retina to cortex.

## 5. Conclusions

The findings shed new light on the conscious perception of ambiguous phenomenal contrast and assimilation in achromatic planar patterns, discussed originally under the premise of Helson’s [8], Beck’s [11], and Heinemann’s [12] studies, and systematically investigated also in terms of Hamada’s “brightness paradox” [9,10,15]. Phenomenal contrast is shown here to be significantly more likely under dark adaptation, while assimilation is more likely in daylight conditions. This is explained by the differential pooling of multiple light/dark ratios by the ON–OFF channels [35,36,37,38]. This pooling affects the relative surface size [34], hence the stronger relative effects of both in the ungrouped conditions, where three spatially separated surfaces are present in the configuration. The relative surface size affects the perceptual principles of grouping underlying the aerial perspective, as discussed in the context of the contrast–depth-cue hypothesis [2,3]. This could, at least partly, explain why adaptation to different lighting conditions affects the luminance contrast–depth-cue hypothesis [2], which predicts physically brighter pattern regions in planar configurations will be perceived as nearer to the conscious observer. This prediction is, as demonstrated here, limited to strong conscious percepts of phenomenal contrast under dark-adapted conditions. These findings could have highly relevant implications for the design of 2D screen and/or virtual reality-based testing of visual spatial abilities in laboratory and clinical settings [48].

## Figures and Tables

**Table 1:** Michelson contrasts (C) of the four pattern configurations, marked as 1, 2, 3, and 4 in Figure 2.

Brighter inducers	
on dark grey background (1):	+0.72
on light grey background (2):	+0.34
Darker inducers	
on dark grey background (3):	+0.40
on light grey background (4):	−0.14

## Data Availability

All the data are available in the manuscript.

## Supplementary Materials

The following supporting information can be downloaded at: https://www.mdpi.com/article/10.3390/brainsci14100966/s1, Original data in terms of the average response probabilities per individual and test condition, provided in the excel table under File S1. File S2 contains the ANOVA output summary reports.
