# Supplementary material for: Environmental Lighting Conditions, Phenomenal Contrast, and the Conscious Perception of Near and Far
Source: Brain Sci. 2024 Sep 26;14(10):966. doi: 10.3390/brainsci14100966 (PMC11505859; doi:10.3390/brainsci14100966)
Supplement: Supplementary file 1 [file brainsci-14-00966-s001.zip › File S2-ANOVA-ProtocolesContrastTask+DepthTask.pdf]

Three Way Analysis of Variance lundi, mars 18, 2024, 17:26:44

Data source: BDL-AR-**ContrastTask**RawData

Balanced Design

Dependent Variable: P"contrast"

**Source of Variation**	**DF**	**SS**	**MS**	**F**	**P**
Adaptation	2	0,891	0,445	20,727	<0,001
Background	1	0,0163	0,0163	0,758	0,401
Configuration	1	0,00586	0,00586	0,273	0,611
Adaptation x Background	2	0,00521	0,00260	0,121	0,887
Adaptation x Configuration	2	0,203	0,102	4,727	0,031
Background x Configuration	1	0,000651	0,000651	0,0303	0,865
Adaptation x Background x Conf	2	0,00521	0,00260	0,121	0,887
Residual	12	0,258	0,0215		
Total	23	1,385	0,0602		

The main effects for Adaptation cannot be properly interpreted since the size of the factor's effect depends upon the level of another factor.

The difference in the mean values among the different levels of Background are not great enough to exclude the possibility that the difference is just due to random sampling variability after allowing for the effects of differences in Adaptation and Configuration. There is not a statistically significant difference (P = 0,401).

The main effects for Configuration cannot be properly interpreted since the size of the factor's effect depends upon the level of another factor.

The effect of different levels of Adaptation does not depend on what level of Background is present. There is not a statistically significant interaction between Adaptation and Background. (P = 0,887)

The effect of different levels of Adaptation depends on what level of Configuration is present. There is a statistically significant interaction between Adaptation and Configuration. (P = 0,031)

The effect of different levels of Background does not depend on what level of Configuration is present. There is not a statistically significant interaction between Background and Configuration. (P = 0,865)

All Pairwise Multiple Comparison Procedures (Holm-Sidak method):
Overall significance level = 0,05

Comparisons for factor: **Background within darkadapted**

**Comparison**	**Diff of Means**	**t**	**Unadjusted P**	**Critical Level**	**Significant?**
dark grey vs. light grey	0,0938	0,905	0,384	0,050	No

Comparisons for factor: **Background within daylight**

**Comparison**	**Diff of Means**	**t**	**Unadjusted P**	**Critical Level**	**Significant?**
dark grey vs. light grey	0,0313	0,302	0,768	0,050	No

Comparisons for factor: **Background within no-rods**

**Comparison**	**Diff of Means**	**t**	**Unadjusted P**	**Critical Level**	**Significant?**
dark grey vs. light grey	0,0313	0,302	0,768	0,050	No

Comparisons for factor: **Adaptation within dark grey**

**Comparison**	**Diff of Means**	**t**	**Unadjusted P**	**Critical Level**	**Significant?**
darkadapted vs. daylight	0,500	4,824	<0,001	0,017	Yes
darkadapted vs. no-rods	0,313	3,015	0,011	0,025	Yes
no-rods vs. daylight	0,188	1,809	0,096	0,050	No

Comparisons for factor: **Adaptation within light grey**

**Comparison**	**Diff of Means**	**t**	**Unadjusted P**	**Critical Level**	**Significant?**
darkadapted vs. daylight	0,438	4,221	0,001	0,017	Yes
darkadapted vs. no-rods	0,250	2,412	0,033	0,025	No

no-rods vs. daylight	0,188	1,809	0,096	0,050	No

Comparisons for factor: **Configuration within darkadapted**

Comparison	Diff of Means	t	Unadjusted P	Critical Level	Significant?
UNGROUPED vs. GROUPED	0,156	1,508	0,158	0,050	No

Comparisons for factor: **Configuration within daylight**

Comparison	Diff of Means	t	Unadjusted P	Critical Level	Significant?
GROUPED vs. UNGROUPED	0,281	2,714	0,019	0,050	Yes

Comparisons for factor: **Configuration within no-rods**

Comparison	Diff of Means	t	Unadjusted P	Critical Level	Significant?
UNGROUPED vs. GROUPED	0,0313	0,302	0,768	0,050	No

Comparisons for factor: **Adaptation within GROUPED**

Comparison	Diff of Means	t	Unadjusted P	Critical Level	Significant?
darkadapted vs. daylight	0,250	2,412	0,033	0,017	No
darkadapted vs. no-rods	0,219	2,111	0,056	0,025	No
no-rods vs. daylight	0,0313	0,302	0,768	0,050	No

Comparisons for factor: **Adaptation within UNGROUPED**

Comparison	Diff of Means	t	Unadjusted P	Critical Level	Significant?
darkadapted vs. daylight	0,688	6,633	<0,001	0,017	Yes
darkadapted vs. no-rods	0,344	3,317	0,006	0,025	Yes
no-rods vs. daylight	0,344	3,317	0,006	0,050	Yes

Comparisons for factor: **Configuration within dark grey**

Comparison	Diff of Means	t	Unadjusted P	Critical Level	Significant?
GROUPED vs. UNGROUPED	0,0417	0,492	0,631	0,050	No

Comparisons for factor: **Configuration within light grey**

Comparison	Diff of Means	t	Unadjusted P	Critical Level	Significant?
GROUPED vs. UNGROUPED	0,0208	0,246	0,810	0,050	No

Comparisons for factor: **Background within GROUPED**

Comparison	Diff of Means	t	Unadjusted P	Critical Level	Significant?
dark grey vs. light grey	0,0625	0,739	0,474	0,050	No

Comparisons for factor: **Background within UNGROUPED**

Comparison	Diff of Means	t	Unadjusted P	Critical Level	Significant?
dark grey vs. light grey	0,0417	0,492	0,631	0,050	No

Power of performed test with alpha = 0,0500: for Adaptation : 1,000
Power of performed test with alpha = 0,0500: for Background : 0,0500
Power of performed test with alpha = 0,0500: for Configuration : 0,0500
Power of performed test with alpha = 0,0500: for Adaptation x Background : 0,0500
Power of performed test with alpha = 0,0500: for Adaptation x Configuration : 0,564
Power of performed test with alpha = 0,0500: for Background x Configuration : 0,0500

Least square means for Adaptation :

Group	Mean
darkadapted	0,766
daylight	0,297
no-rods	0,484

Std Err of LS Mean = 0,0518

Least square means for Background :

Group	Mean
dark grey	0,542
light grey	0,490

Std Err of LS Mean = 0,0423

Least square means for Configuration :

Group	Mean
GROUPED	0,531
UNGROUPED	0,500

Std Err of LS Mean = 0,0423

Least square means for Adaptation x Background :

Group	Mean
darkadapted x dark grey	0,813
darkadapted x light grey	0,719
daylight x dark grey	0,313
daylight x light grey	0,281
no-rods x dark grey	0,500
no-rods x light grey	0,469

Std Err of LS Mean = 0,0733

Least square means for Adaptation x Configuration :

Group	Mean
darkadapted x GROUPED	0,688
darkadapted x UNGROUPED	0,844
daylight x GROUPED	0,438
daylight x UNGROUPED	0,156
no-rods x GROUPED	0,469
no-rods x UNGROUPED	0,500

Std Err of LS Mean = 0,0733

Least square means for Background x Configuration :

Group	Mean
dark grey x GROUPED	0,563
dark grey x UNGROUPED	0,521
light grey x GROUPED	0,500
light grey x UNGROUPED	0,479

Std Err of LS Mean = 0,0598

Least square means for Adaptation x Background x Conf :

Group	Mean
darkadapted x dark grey x G	0,750
darkadapted x dark grey x U	0,875
darkadapted x light grey x	0,625
darkadapted x light grey x	0,813
daylight x dark grey x GROUPED	0,438
daylight x dark grey x U	0,188
daylight x light grey x	0,438
daylight x light grey x	0,125
no-rods x dark grey x GROUPED	0,500
no-rods x dark grey x U	0,500
no-rods x light grey x GROUPED	0,438
no-rods x light grey x	0,500

Std Err of LS Mean = 0,104

Three Way Analysis of Variance jeudi, mars 14, 2024, 17:58:18

Data source: BDL-AR-**Depth**Task

Balanced Design

Dependent Variable: P"nearer"

Source of Variation	DF	SS	MS	F	P
Adaptation	2	0,693	0,346	12,667	0,001
Background	1	0,0104	0,0104	0,381	0,549
Configuration	1	0,375	0,375	13,714	0,003
Adaptation x Background	2	0,0677	0,0339	1,238	0,324
Adaptation x Configuration	2	0,109	0,0547	2,000	0,178
Background x Configuration	1	0,0417	0,0417	1,524	0,241
Adaptation x Background x Conf	2	0,0208	0,0104	0,381	0,691

Residual	12	0,328	0,0273
Total	23	1,646	0,0716

The difference in the mean values among the different levels of Adaptation are greater than would be expected by chance after allowing for the effects of differences in Background and Configuration. There is a statistically significant difference (P = 0,001). To isolate which group(s) differ from the others use a multiple comparison procedure.

The difference in the mean values among the different levels of Background are not great enough to exclude the possibility that the difference is just due to random sampling variability after allowing for the effects of differences in Adaptation and Configuration. There is not a statistically significant difference (P = 0,549).

The difference in the mean values among the different levels of Configuration are greater than would be expected by chance after allowing for the effects of differences in Adaptation and Background. There is a statistically significant difference (P = 0,003). To isolate which group(s) differ from the others use a multiple comparison procedure.

The effect of different levels of Adaptation does not depend on what level of Background is present. There is not a statistically significant interaction between Adaptation and Background. (P = 0,324)

The effect of different levels of Adaptation does not depend on what level of Configuration is present. There is not a statistically significant interaction between Adaptation and Configuration. (P = 0,178)

The effect of different levels of Background does not depend on what level of Configuration is present. There is not a statistically significant interaction between Background and Configuration. (P = 0,241)

All Pairwise Multiple Comparison Procedures (Holm-Sidak method):
Overall significance level = 0,05

Comparisons for factor: **Adaptation**

**Comparison**	**Diff of Means**	**t**	**Unadjusted P**	**Critical Level**	**Significant?**
darkadapted vs. no-rods	0,375	4,536	<0,001	0,017	Yes
darkadapted vs. daylight	0,344	4,158	0,001	0,025	Yes
daylight vs. no-rods	0,0313	0,378	0,712	0,050	No

Comparisons for factor: **Configuration**

**Comparison**	**Diff of Means**	**t**	**Unadjusted P**	**Critical Level**	**Significant?**
UNGROUPED vs. GROUPED	0,250	3,703	0,003	0,050	Yes

Power of performed test with alpha = 0,0500: for Adaptation : 0,978
Power of performed test with alpha = 0,0500: for Background : 0,0500
Power of performed test with alpha = 0,0500: for Configuration : 0,918
Power of performed test with alpha = 0,0500: for Adaptation x Background : 0,0778
Power of performed test with alpha = 0,0500: for Adaptation x Configuration : 0,179
Power of performed test with alpha = 0,0500: for Background x Configuration : 0,0973

Least square means for Adaptation :

**Group**	**Mean**
darkadapted	0,969
daylight	0,625
no-rods	0,594

Std Err of LS Mean = 0,0585

Least square means for Background :

**Group**	**Mean**
dark grey	0,750
light grey	0,708

Std Err of LS Mean = 0,0477

Least square means for Configuration :

**Group**	**Mean**
GROUPED	0,604
UNGROUPED	0,854

Std Err of LS Mean = 0,0477

Least square means for Adaptation x Background :

Group	Mean
darkadapted x dark grey	0,969
darkadapted x light grey	0,969
daylight x dark grey	0,719
daylight x light grey	0,531
no-rods x dark grey	0,563
no-rods x light grey	0,625

Std Err of LS Mean = 0,0827

Least square means for Adaptation x Configuration :

Group	Mean
darkadapted x GROUPED	0,938
darkadapted x UNGROUPED	1,000
daylight x GROUPED	0,469
daylight x UNGROUPED	0,781
no-rods x GROUPED	0,406
no-rods x UNGROUPED	0,781

Std Err of LS Mean = 0,0827

Least square means for Background x Configuration :

Group	Mean
dark grey x GROUPED	0,667
dark grey x UNGROUPED	0,833
light grey x GROUPED	0,542
light grey x UNGROUPED	0,875

Std Err of LS Mean = 0,0675

Least square means for Adaptation x Background x Conf :

Group	Mean
darkadapted x dark grey x G	0,938
darkadapted x dark grey x U	1,000
darkadapted x light grey x	0,938
darkadapted x light grey x	1,000
daylight x dark grey x GROUPED	0,625
daylight x dark grey x U	0,813
daylight x light grey x	0,313
daylight x light grey x	0,750
no-rods x dark grey x GROUPED	0,438
no-rods x dark grey x U	0,688
no-rods x light grey x GROUPED	0,375
no-rods x light grey x	0,875

Std Err of LS Mean = 0,117
